# Effects of Buoyancy and Surface Roughness on Mechanical Characterization of Shipbuilding Steel by Immersion Instrumented Indentation

**DOI:** 10.3390/mi17070766

**Published:** 2026-06-24

**Authors:** Xiaoyuan Zhang, Zhongyu Zhao, Zhaoxin Wang, Shuai Li, Chao Sun, Yan Xia, Zhanqiang Liu, Yukui Cai, Bing Wang, Shunbo Wang

**Affiliations:** 1 School of Mechanical Engineering, Shandong University, Jinan 250061, Chinamelius@sdu.edu.cn (Z.L.); caiyukui@sdu.edu.cn (Y.C.); sduwangbing@sdu.edu.cn (B.W.); 2 State Key Laboratory of Advanced Equipment and Technology for Metal Forming, Shandong University, Jinan 250061, China; 3 Key Laboratory of High Efficiency and Clean Mechanical Manufacture of Ministry of Education and National Demonstration Center for Experimental Mechanical Engineering Education at Shandong University, Jinan 250061, China; 4 Shandong North Coastal Machinery Co., Ltd., Zibo 255200, China; 15550327237@163.com (S.L.); 17560308839@163.com (C.S.); 5 Shandong Machinery Design & Research Institute, Jinan 250031, China; yxia@qlu.edu.cn; 6 School of Airspace Science and Engineering, Shandong University, Weihai 264209, China; 7 School of Mechanical & Aerospace Engineering, Jilin University, Changchun 130025, China; wangshunbo@jlu.edu.cn

**Keywords:** immersion instrumented indentation, surface roughness, buoyancy effect, indentation responses, finite element simulations

## Abstract

To develop the applications of nanoindentation in a liquid environment, a higher requirement has been presented for evaluating measured errors of micromechanical performance, particularly for the immersion indentation. In this study, a numerical investigation was conducted to explore the influence of buoyancy and surface roughness on the indentation responses of EH36 steel. The results show that a difference in indentation load–depth curves and mechanical properties with and without the buoyancy effect is observed as the indentation depth increases. The relative calculated errors of reduced modulus and indentation hardness are more than 25.35% and 1.92%, respectively. Meanwhile, as the surface roughness increases, a pronounced increase in the data scatter of indentation responses is observed, especially at shallow nanoindentations. The coupling effects of buoyancy and surface roughness on the deviation of indentation data exhibit a competitive relationship rather than a simple cumulative effect. The measured errors of reduced modulus at roughness values below 0.10 μm are predominantly affected by buoyancy during immersion indentations, while the surface roughness is the main factor in other cases. This study provides a comprehensive understanding of these factors and predicts the qualitative variations in micromechanical responses under assumed immersed conditions.

## 1. Introduction

High-strength steels have been widely used in manufacturing ship structures due to their excellent mechanical properties [1,2]. Under conditions of equivalent structural strength, the utilization of high-strength steels (e.g., EH36) offers the advantages of reduced hull weight, increased carrying capacity, and slightly lower operational costs. However, for marine engineering structures, superior corrosion and fatigue resistance is required, particularly in the welded joint zones [3,4]. The macroscopic damages often originate from micro-defects on the surface or subsurface of engineering structures and result in a degradation of micromechanical properties. Accordingly, the accurate assessment of structural integrity and residual service life necessitates the convenient evaluation of mechanical performance at micro- and nanoscopic scales.

Instrumented indentation testing (IIT) has been used as a semi-nondestructive method to determine the local mechanical properties of bulk materials. For instance, Pamnani et al. established the correlation between microstructure, microhardness, and tensile properties obtained using automated ball indentation [5]. Nathan et al. evaluated the microhardness evolution in the weld metal regions of high-strength steel joints fabricated by friction stir welding and fusion welding [6]. Meanwhile, IIT has been successfully extended to the investigation of elastic-plastic properties in biological tissues [7], electrochemical and corrosion behaviors of metal components [8,9]. Nevertheless, in practical applications, indentation measurements are highly susceptible to the surface condition of materials, which affects the measured errors of the true mechanical properties of materials.

On the one hand, when nanoindentation experiments are performed under immersed conditions, the indenter is inevitably subjected to the buoyancy, resulting in inaccuracies in the measurement of the indentation load. Currently, several research institutions and commercial companies have successively immersion-instrumented indentation apparatus. Zhao et al. developed a novel low-temperature indentation apparatus that cools with Peltier coolers [10]. To minimize the contact thermal drift, both the specimen and the indenter tip are immersed in 50% methanol–water solution. Zhao et al. examined the effects of atmospheric conditions, electrolyte composition, structural degradation, and electrode volume expansion on lithium reactions using a nanoindentation integrated with a custom fluid cell in an inert environment [11,12]. Meanwhile, Bruker Inc., as an example, has introduced an in situ electrochemical environment loading cell [13,14]. However, the aforementioned studies did not thoroughly investigate the floating effect caused by the fluid media in immersion indentation, and the measured indentation load–depth (*P*-*h*) curves were not corrected. Accordingly, this may introduce extra measurement errors, particularly at the micro- and nanoscale.

On the other hand, the surface quality could progressively deteriorate with increasing corrosion time due to the chemomechanical effects. Several studies have proven that there is a significant effect of surface roughness on indentation responses, such as indentation depth and contact area. Chen et al. evaluated roughness effects via sinusoidal surface profiles to correct constitutive parameters of soft polymers. A plane strain modeling assumption is preferred, owing to a lack of axisymmetry for real surface topography [15]. Nevertheless, this FE model employs a wedge indenter, making it difficult to obtain accurate *P*-*h* curves and micromechanical properties. Campbell et al. demonstrated that random surface roughness significantly affected plastic zone development and residual stress distribution via finite-element (FE) nanoindentation simulations [16]. Tan et al. revealed that the indentation hardness of a nickel-based superalloy IN718 decreases with the increase in surface roughness [17]. These investigations indicate that surface roughness serves as a significant contribution to measurement uncertainty and data variability in IIT, primarily through its influence on the distribution of local contact stress.

As computational science has advanced rapidly, FE modeling has been widely employed to evaluate the mechanical response during indentation. Influence factors like the indenter tip rounding defect [18], contact thermal drift [19], pore densification [20], substrate effect [21], etc., are investigated for the results of indentation tests via FE simulations. However, few studies assessed the floating effect during immersion instrumented indentations. Meanwhile, in practical immersion IIT experiments for estimating the corrosion behaviors of high-strength steels, the buoyancy and surface roughness in the indentation area are difficult to determine and eliminate. To more accurately evaluate the indentation responses under in-service conditions, these coupled topographical and environmental factors can be interpreted within the framework of surface integrity and data variance standardization [22].

In this paper, a numerical investigation was conducted to explore the influence of buoyancy and surface roughness on the indentation responses of EH36 steel. Two kinds of typical immersed conditions and several rough surfaces were discussed for comparative studies of *P*-*h* curves and indentation responses in conventional IIT and immersion instrumented indentation. This paper aims to understand the numerical sensitivities of error margins associated with the indentation of EH36 steel in a liquid environment and to assess their impact on the estimation of material performance. A comprehensive understanding of the aforementioned factors could enhance IIT applications to accurately predict the influence of environmental and topographic factors.

## 2. Theory and Modeling

### 2.1. Immersion Indentation

**Figure 1:**
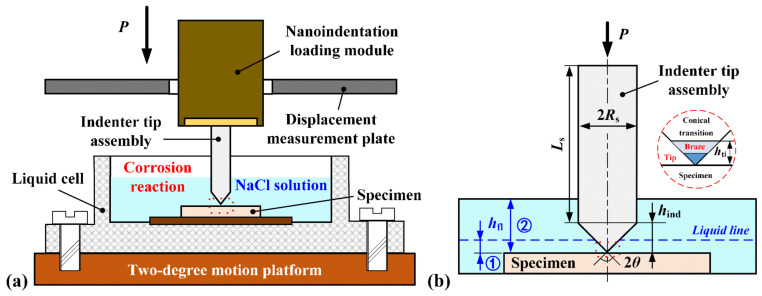
(**a**) A schematic representation of the immersion indentation apparatus. (**b**) The simplified 2D structural schematic of the experiment model. The liquid line is shown as a blue dashed line, and the locally magnified region around the indenter tip is marked by a red dashed circle. ① and ② denote the immersion depths for the cases in which the liquid line is located at the indenter tip and at the shaft, respectively.

A schematic representation of the immersion indentation apparatus is presented in Figure 1a. The high-strength steel specimen is securely mounted onto the specimen holder. Under the assumption that the floating effect on the specimen is negligible, only the indenter is the component subjected to the buoyancy force throughout the experimental procedure. During the immersion indentation process, the progressive downward movement of the indenter results in its exposure to buoyancy immediately upon liquid surface contact. As the immersion depth (*h*_i_) increases, the buoyancy acting on the specimen increases correspondingly throughout the entire immersion process. Hence, inaccurate measurement of the reaction force on the indenter can result in an overestimation of the indentation load in immersion indentation compared to traditional indentation. It is of great significance to determine the influence of buoyancy on *P*-*h* curves using a numerical approach.

Herein, we take a commercial Berkovich indenter from Synton-MDP Inc., Nidau, Switzerland, as an example. It was employed in our previous series of self-custom indentation apparatuses [23,24,25]. The indenter tip assembly consists of a shaft and a geometrically defined tip, which are joined by a titanium-based brazing alloy. It is noted that the indenter tip and the brazing layer have dimensions (*h*_ti_) on the order of several tens of micrometers, such as *h*_ti_ = 60 μm [19]. As a result, the indenter tip tends to be fully immersed in the liquid. The cross-sectional schematic of the immersion indentation model shows the liquid-phase line after the indenter contacts the specimen surface under two different conditions, as indicated by the dashed lines in Figure 1b. The measured indentation depth is defined as *h*, and the symbol *h*_fl_ is used to represent the immersion depth of the specimen. *h*_ind_ represents the height of the indenter tip assembly, which comprises a conical transition region with the half-cone angle of *θ* (≈15°) between the clamping shank and the indenter tip. Meanwhile, the radius of the indenter shaft is *R*_s_, and its height is *L*_s_. Consequently, the equation of immersion depth (*h*_i_) can be expressed as:

(1) $$h_{i}=h+h_{\mathrm{fl}}$$

where the value of hi can be classified into two different regimes, namely, $h_{i}\leq h_{\mathrm{ind}},\;\mathrm{and}\;h_{i}>h_{\mathrm{ind}}$.

Two assumptions are proposed in this study: (1) the fluid remains relatively static during the indentation process; (2) the surface tension of the liquid media adhered to the indenter is negligible [24]. Regarding the Berkovich indenter, the indentation contact area (*A*_c_) without tip roundness is approximately $24.56h_{c}^{2}$, and *h*_c_ denotes the indentation contact depth.

In the case of $h_{i}\leq h_{\mathrm{ind}}$, the equation of buoyancy can be expressed as:

(2) $$P_{\mathrm{fl}}=\rho g\left[\int_{h_{\mathrm{ti}}}^{h_{i}}\pi \left[R_{1}^{2}+2R_{1}z\frac{R_{s}-R_{1}}{H}+z^{2}{\left(\frac{R_{s}-R_{1}}{H}\right)}^{2}\right]dz+\sqrt{3}h_{\mathrm{ti}}^{3}\tan^{2}\alpha \right]$$

(3) $$R_{1}=2h_{\mathrm{ti}}\tan\alpha$$

(4) $$H=\frac{R_{s}-R_{1}}{\tan\theta }$$

where *P*_fl_ denotes the buoyancy during the immersion indentation procedure, *ρ* denotes the density of immersion liquid, *g* denotes the gravitational acceleration, and *H* denotes the height of the conical transition region. Meanwhile, *R*_1_ is the radius of the circumscribed circle at the Berkovich tip, and *z* denotes the height of liquid line.

Similarly, the equation of buoyancy in the case of $h_{i}>h_{\mathrm{ind}}$ can be expressed as:

(5) $$P_{\mathrm{fl}}=\rho g\left[\int_{h_{\mathrm{ind}}}^{h_{i}}\pi R_{s}^{2}dz+\frac{1}{3}\pi H\left(R_{1}^{2}+R_{1}R_{s}+R_{s}^{2}\right)+\sqrt{3}h_{\mathrm{ti}}^{3}\tan^{2}\alpha \right]$$

### 2.2. Immersion Indentation Modeling for the Rough Surface

**Figure 2:**
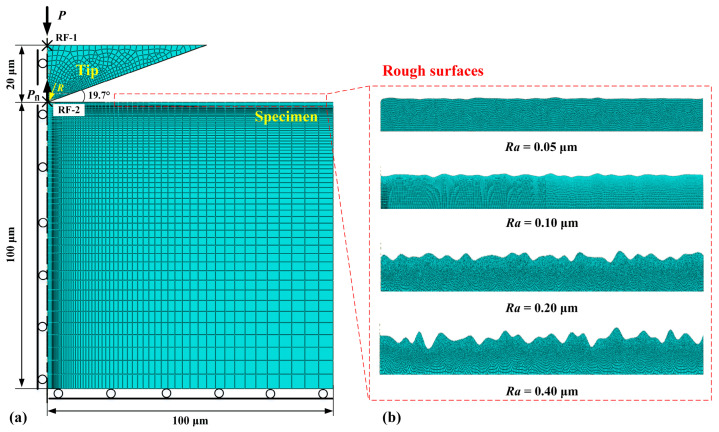
(**a**) 2D axisymmetric FE model for immersion indentation. (**b**) 2D rough surface models with different Ra values.

A two-dimensional (2D) FE model was developed using ABAQUS/Standard 2020 software to simulate the indentation of EH36 high-strength steel, as shown in Figure 2a. According to our previous studies [19,26,27], the geometry and dimensions of each component in the indenter tip assembly were configured to simulate the experimental indentation process. Herein, to improve computational efficiency, the indenter tip assembly was simplified as a conical tip with a half angle of 70.3° and a blunt radius (*R*) of ~72.83 nm [18]. It can yield the same projection area as the Berkovich indenter. Although numerous studies have reported that the axisymmetric indenter assumption is used to investigate the effect of rough surfaces on indentation responses [28,29,30], it is admittedly insufficient for exact quantitative replication of three-dimensional localized stress fields on stochastic surfaces. When the surface roughness amplitude is on the same order of magnitude as the indentation depth, the 2D FE model remains highly capable of capturing the contact interactions between the indenter profile and random surface profile. The results also indicate the drastic increase in data scatter at shallow indentation depths, which is in good agreement with the conclusions in the previous literature [31,32]. Therefore, the FE simulation results can be utilized to extract *P*-*h* curves and mechanical properties.

The indenter was set as a perfectly elastic body with a height of 20 μm. The elastic modulus (*E*_i_) was 1140 GPa, and the Poisson’s ratio (*υ*_i_) was set to 0.07 [33]. The indenter assembly shaft has a radius of 1.25 mm and a height of 15 mm. Meanwhile, the specimen was modeled with dimensions of 0.1 mm × 0.1 mm. The stress–strain curve (*σ*-*ε*) for the EH36 steel specimen can be described by the following equation:

(6) $$\left\{\begin{array}{c} \sigma =E\varepsilon ,\;\mathrm{for}\;\varepsilon \leq \frac{\sigma_{y}}{E}\\ \sigma =K\varepsilon^{n}=E\varepsilon^{n}{\left(\frac{\sigma_{y}}{E}\right)}^{1-n},\;\mathrm{for}\;\varepsilon >\frac{\sigma_{y}}{E} \end{array}\right.$$

where *K* represents the strength coefficient, *σ*_y_ represents the yield strength, and *n* represents the work-hardening exponent, respectively. Accordingly, the elastic modulus (*E*) of the specimen was set to be 206 GPa, with a yield strength of 432 MPa, and the values of *n* and Poisson’s ratio (*υ*) were 0.1632 and 0.3, respectively [34,35].

All components in the model were meshed with 8-node linear hexahedral reduced integration elements (CAX4R). Local mesh refinement was employed in the contact region, with a gradual transition to a coarser mesh farther away. A surface-to-surface contact pair was defined between the indenter (master surface) and the specimen (slave surface). Moreover, normal behavior was governed by a hard contact constraint, while tangential behavior was modeled as the Coulomb friction coefficient of 0.1 [18,36]. The boundary conditions were prescribed in accordance with the experimental indentation procedure: the bottom surface of the specimen was constrained in *X*-axis degree of freedom, while the lateral and top surfaces remained free. Meanwhile, the indenter could only move along the *Y*-axis.

Displacement control was employed for the whole indentation process, imposing maximum indentation depths (*h*_m_) of 100, 200, 300, 400, and 500 nm, respectively. In immersion indentation, the indenter experiences an extra buoyancy force that increases with the value of *h*_m_. Consequently, an equivalent buoyancy load opposite to the indentation direction is applied to the reference point on the indenter end. In this paper, to simulate the shipbuilding steel plate under marine corrosion environment, the homogeneous liquid medium was assumed to be 3.5% NaCl, with a density of 1.02 g/cm^3^ [37]. Furthermore, the submergence depths of the indenter at the onset of contact are assumed to be 2 mm and 5 mm for the aforementioned two immersion cases. The maximum value of *P*_fl_ is around 0.14 mN calculated from Equations (2)–(5).

Rough surface models were directly generated in ABAQUS to assess the effect of surface roughness on the mechanical response of EH36 steel under immersion indentation. Herein, based on the RufGen plugin developed by Lim et al. [38], the initial rough surface was established by the subroutine of the height distribution method to modify the *Y*-coordinates (*Y*(*x*)) of each node. The probability density function (PDF) of the rough surface follows a Gaussian distribution. *Ra* is defined as the arithmetic mean of the absolute values of the *Y*-coordinates of the nodes in the sampling line (*L*_x_), as follows:

(7) $$Ra=\frac{1}{L_{x}}\int_{0}^{L_{x}}\left|Y\left(x\right)\right|dx$$

The representative rough surface models (e.g., *Ra* = 0.05 μm, 0.10 μm, 0.20 μm, and 0.40 μm) are shown in Figure 2b. Meanwhile, a smooth surface was set as the reference group. A total of 20 simulations were conducted for each value of surface roughness, with indentation locations randomly distributed across the rough surfaces. To assess the statistical characteristics of the detection data, the standard deviation of independent repeated measurements was employed for the error bars.

## 3. Results and Discussions

### 3.1. Effect of Buoyancy on P-h Curves and Mechanical Properties

To avoid the effect of mesh refinement on the micromechanical properties prior to the parametric analysis, a convergence study of the FE simulations has been conducted. Herein, the influence of various refined mesh sizes (e.g., 27.5 nm, 25 nm, 20 nm, 15 nm, and 12.5 nm) on the deviation of curvature (*c*_2_) in the loading segment is investigated. The relative error of curvature (*η*) can be calculated as [18]:

(8) $$\eta =\frac{\left|c_{2}^{n+1}-c_{2}^{n}\right|}{\left|c_{2}^{n+1}\right|}$$

where $c_{2}^{n+1}\;,\;c_{2}^{n}$ denote the values of reduced modulus at the (*n* + 1)th and *n*th iterations.

**Figure 3:**
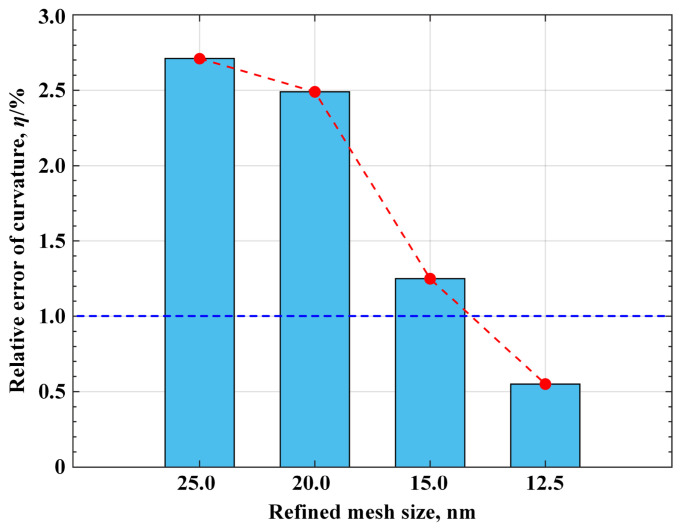
The convergence study for FE simulations with different mesh sizes. The blue dashed line represents the 1% relative error threshold, while the red dashed line indicates the trend of error variation with different refined mesh sizes.

In general, when *η* < 1%, the simulation results are defined as converged [18]. As shown in Figure 3, the value of *η* is only 0.55% for the refined mesh size of 12.5 nm. Therefore, the refined mesh size of our FE simulation model is reasonable.

**Figure 4:**
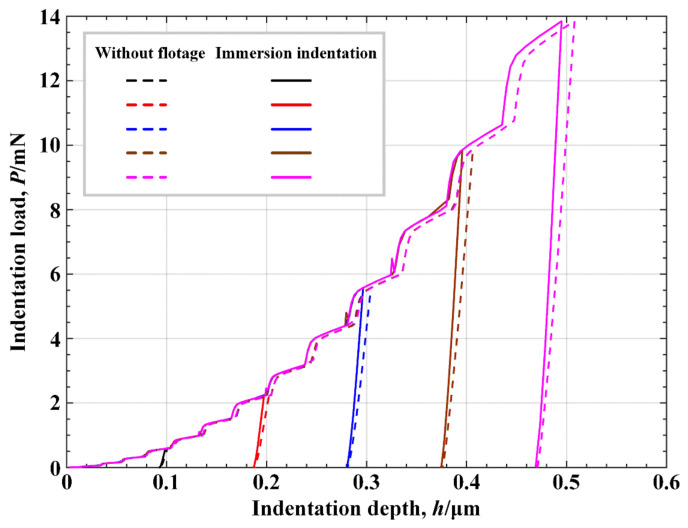
Representative *P*-*h* curves for the immersion indentation on the smooth surface with or without the floating effect.

A comparable study was conducted to investigate the effect of buoyancy on indentation responses, with maximum indentation depths varying from 100 nm to 500 nm. Figure 4 shows representative *P*-*h* curves for the immersion indentation on the smooth surface with or without the floating effect. The loading curve exhibits oscillatory fluctuations that cannot be attributed to the discreteness of the data, given that each curve comprises several hundred data points. These oscillations are also observed, albeit with reduced amplitude, at lower indentation loads. It could be caused by the severe localized plastic deformation under the sharp indenter tip, requiring continuous severe discontinuity iterations to resolve the contact boundary. In other words, the contact chattering creates high-frequency, low-amplitude numeric force oscillations during indentation displacement-controlled loading. The oscillations on the loading segment have been observed in numerous previous studies, such as Li et al. [39], Niu et al. [40], and Karbasian et al. [41]. Notably, although the contact stress under the spherical indenter is smoother than that of the sharp indenter, a similar oscillation phenomenon in the loading segment is still observed. As shown in Figure 4, the unloading curves exhibit exceptional smoothness without any noticeable oscillations. The critical mechanical properties are extracted from the unloading segment of the *P*-*h* curves using the Oliver–Pharr method. Herein, the simulated reference group of smooth surfaces calculated an average reduced modulus of 205.53 GPa, which perfectly matches the input value with a negligible relative error of only 0.23%. Therefore, both the indentation hardness and reduced modulus can be determined from the unloading segment of the *P*-*h* curves.

The results in Figure 4 demonstrate that indentation loads at the same indentation depth during the immersion indentation are larger than those obtained from the conventional indentation without the floating effect. It could be attributed to the fact that the buoyancy acts in the opposite direction to the indentation. In addition, it can be clearly seen that the slope of the unloading segment (*S* = d*P*/d*h*, i.e., the contact stiffness) in immersion indentation significantly increases. Therefore, both the loading and unloading segments of the *P*-*h* curves deviate from their conventional indentation to some extent.

Herein, the indentation hardness and reduced modulus can be calculated using the Oliver–Pharr method. To quantitatively interpret how buoyancy affects the immersion indentation results, the relative errors of reduced modulus and indentation hardness (*ε*_E_, *ε*_H_) can be calculated using the following equation:

(9) $$\left\{\begin{array}{c} \varepsilon_{E}=\frac{E_{\mathrm{fl}}-E_{0}}{E_{\mathrm{fl}}}\times 100\%\\ \varepsilon_{H}=\frac{H_{\mathrm{fl}}-H_{0}}{H_{\mathrm{fl}}}\times 100\% \end{array}\right.$$

where *E*_fl_ and *H*_fl_ are the reduced modulus and indentation hardness obtained from immersion indentation. *E*_0_ and *H*_0_ are the results obtained from conventional indentation without the floating effect.

**Figure 5:**
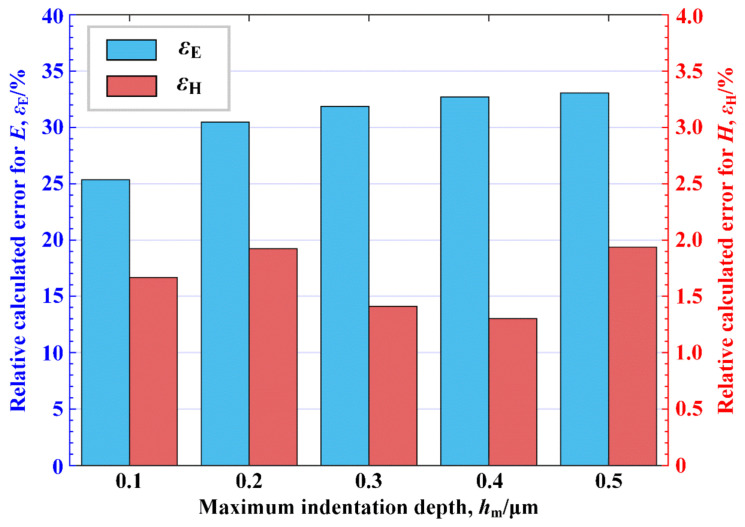
Relative calculated errors of reduced modulus and indentation hardness for the conventional indentation compared with immersion indentations with different values of *h*_m_.

As shown in Figure 5, the values of *ε*_E_ gradually increase from 25.35% to 33.06% with an increase in the maximum indentation depth. Meanwhile, the values of *ε*_H_ are approximately 1.92% with different maximum indentation depths. Compared with the previous study for various biological tissues, such as the bone, brain, etc. [42], the calculated errors obviously decrease owing to the higher indentation loads at the same value of *h*_m_. The results in Figure 5 indicate that the buoyancy has a significant effect on the reduced modulus, whereas the calculated error for the indentation hardness is independent of the indentation depth and can be negligible.

### 3.2. Effect of Surface Roughness on P-h Curves and Mechanical Properties

**Figure 6:**
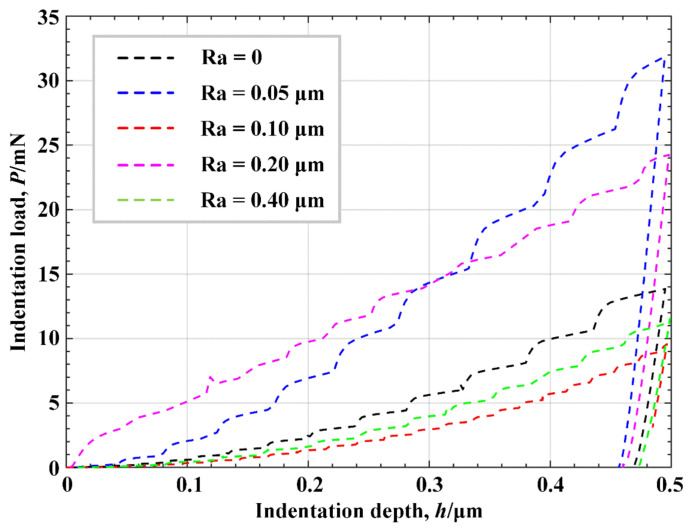
The representative simulated *P*-*h* curves for various surface roughness (*Ra* = 0.05–0.4 μm) and the reference group of smooth surfaces (*Ra* = 0).

To quantify the contribution of surface roughness, four rough surface models (*Ra* = 0.05–0.4 μm) were compared against a perfectly smooth reference model in terms of indentation responses. Figure 6 illustrates the representative *P*-*h* curves (*h*_m_ = 500 nm) between the different roughness models and the ideal smooth surface model. It is obvious that the *P*-*h* curves exhibit significant scatter due to the surface roughness effect, such as the curve obtained with a roughness value of 0.20 μm. Therefore, it also constitutes one of the primary reasons for calculated error in the analysis of indentation data [43,44]. In other words, surface roughness markedly affects the scatter of indentation responses. This result is consistent with the findings of numerous previous studies [17,45].

**Figure 7:**
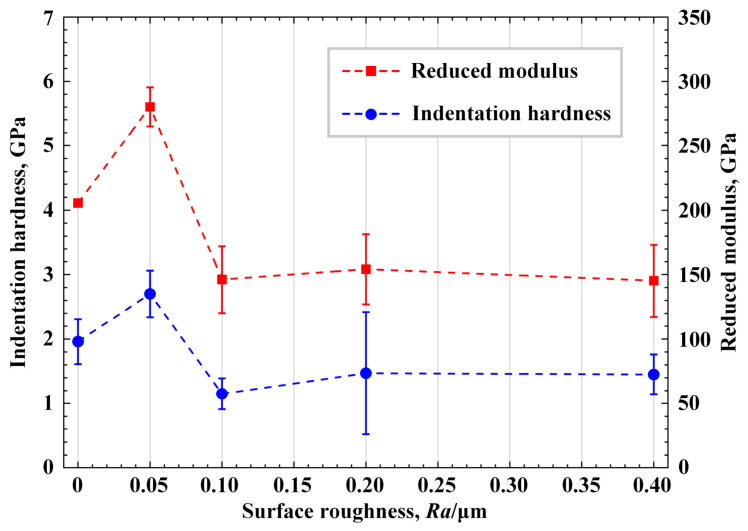
The reduced modulus and indentation hardness obtained from the simulated *P*-*h* curves at different *Ra* values of surface roughness.

In Figure 7, the mean values and standard deviation of reduced modulus and indentation hardness are calculated from each *P*-*h* curve at different *Ra* values of surface roughness. The results indicate that there is the lowest scatter of indentation data for the reference group, namely, the smooth surface model. Furthermore, it can be observed that although the roughness of the rough surface model is as low as *Ra* = 0.05 μm, the calculated error for the elastic modulus compared with the reference value reaches ~36.31%, and that for the indentation hardness reaches ~37.76%. As the surface roughness increases, the standard deviation of the measured data remains almost unchanged (<20%), while the relative errors exhibit considerable scatter, indicating that the factors influencing indentation tests on rough surfaces are not solely related to surface roughness. At low roughness, the indentation response is dominated by local topological geometric confinement among asperities, resulting in an apparent overestimation of mechanical properties. Conversely, as the ratio of *Ra* to *h*_m_ increases, the discontinuous plastic collapse of isolated asperities leads to the degradation of the true contact area; that is, the mechanical properties exhibit macroscopic softening features.

Based on the Oliver–Pharr model, the indentation results mainly depend on the indentation contact area and maximum indentation load (*P*_m_). Compared with the smooth surface model, the actual area of initial contact on rough surfaces is limited to the peaks of the highest asperities, which are composed of many micro-asperities and valleys. Rough surfaces exhibit a complex and irregular morphology that is often characterized as random. Consequently, the material exhibits an apparently higher hardness when the indentation site is located in a valley, whereas indentation on a peak yields a more compliant response. This topography dependence is intrinsically governed by the alteration of the localized plastic zone and the real contact area. The plastic zone within the valley could be constrained, and the subsurface hydrostatic stress fields are modified. In contrast, indentation on sharp asperities and peak contacts drastically enlarges *A*_c_. This leads to higher measured values and a significant increase in random errors, which is in good agreement with classic contact mechanics simulations. Meanwhile, in this study, the indentation depth is on the same order as the characteristic length scale of the surface roughness. The hardening effect of surface asperities becomes more pronounced, leading to higher measured values and a significant increase in random errors [46].

**Figure 8:**
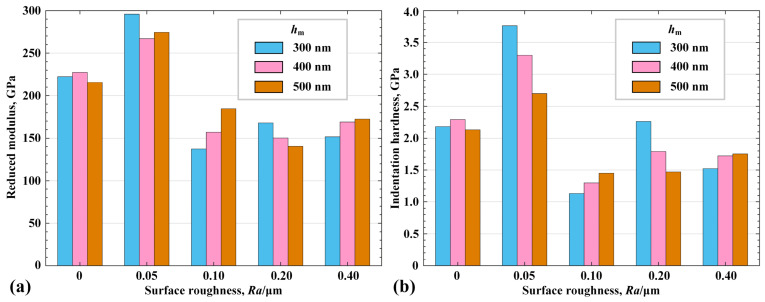
The variations in reduced modulus (**a**) and indentation hardness (**b**) with different surface roughness at various maximum indentation depths.

Figure 8 shows the variations in reduced modulus and indentation hardness with different surface roughness at various maximum indentation depths of 300 nm, 400 nm, and 500 nm, respectively. The results indicate that for the smooth surface model, the reduced modulus and indentation hardness are independent of the indentation depth. As the value of *h*_m_ increases for rough surface models, the measured results gradually trend towards the reference values obtained from the smooth surface model; i.e., the influence of roughness progressively weakens. Accordingly, it can be guaranteed that enough indentation depth is chosen to avoid the surface roughness effect, such as more than 20 times the *Ra* values [47].

### 3.3. Coupling Effects of Buoyancy and Surface Roughness

In practical indentation in the liquid environment, the buoyancy and surface roughness are inherently coupled. However, it is misleading to attribute variations in indentation responses only to contact conditions, particularly in FE simulations. Herein, this study integrates both buoyancy and surface roughness effects into the analysis of indentation responses.

**Figure 9:**
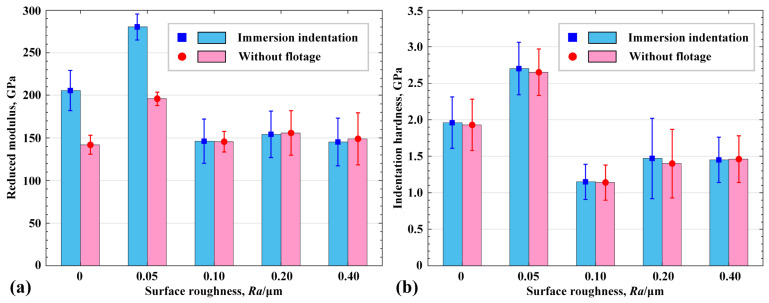
The variation in reduced modulus (**a**) and indentation hardness (**b**) with the combined influence of buoyancy and surface roughness.

Figure 9 presents the coupling effects of buoyancy and surface roughness on the reduced modulus and indentation hardness during immersion indentations. On the one hand, when the *Ra* value of the rough surface is less than 0.05 μm, the relative errors of reduced modulus are 30.96% for the smooth surface models and 30.26% for the rough surface model with an *Ra* value of 0.05 μm, owing to the buoyancy effect. As the roughness increases, the buoyancy effect gradually weakens, and the influence of surface roughness becomes more significant. Meanwhile, the scatter in the measured results increases slightly.

On the other hand, the results in Figure 9b show that the values of indentation hardness between immersion indentation and conventional indentation without the buoyancy, as well as their standard deviation, are nearly the same. Therefore, it could mainly be affected by the surface roughness effect rather than the floating effect. Overall, the coupling effect of buoyancy and surface roughness reveals that surface roughness is the principal contributor to variations in micromechanical properties, with buoyancy playing only a minor role. This phenomenon aligns with the underlying competition mechanism observed in multi-factor surface integrity assessments, where dominate mechanical or geometric factors overshadow minor environment alterations [22].

It is worth noting that capillary effects and surface tension forces (*F*_st_) acting on the indenter shaft cannot be ignored from an absolute force perspective at the micro- and nano-scale. An order-of-magnitude estimation indicates that for an indenter shaft radius of 1.25 mm in a 3.5% NaCl solution, the maximum vertical surface tension force is approximately 0.15 mN. It is almost comparable in magnitude to the buoyancy force. However, since the maximum indentation depth is only at the micrometer- or nanometer-scale, the liquid meniscus remains stable, rendering *F*_st_ a constant background force during the quasi-static indentation cycle. Meanwhile, mechanical properties are calculated from the different unloading responses such that this constant force offset does not alter the data scatter or the stiffness deviations investigated herein. Notably, as with our previous custom-designed indentation apparatus [10], this constant capillary force is typically eliminated via pre-contact loading calibration.

Furthermore, it is instructive to discuss the extensibility of the current numerical results to other fluid environments. While the specific numerical values are bound to EH36 steel in a 3.5% NaCl solution, the empirical relationships and competitive mechanisms could remain universally applicable. For softer metals where the absolute indentation loading is low, the relative distortion induced by the buoyancy force on the unloading slope would be more pronounced, yielding higher evaluation errors for the reduced modulus. In contrast, for exceptionally hard metals, this floating effect becomes less significant. Meanwhile, substituting the medium with denser fluids (e.g., drilling mud) would linearly upscale the buoyancy magnitude according to Equations (2)–(5), thereby amplifying the reduced modulus deviations.

While the numerical results presented in this study provide a quantitative approach for understanding buoyancy and surface roughness effects, translating these simulation results into practical experiments involves several inherent challenges and limitations. First, since conventional nanoindentation tests are typically quasi-static loading and unloading procedures, only a nearly constant surface tension and a uniformly varying buoyancy force were taken into account. However, the transiently dynamic resistance and liquid adhesion exerted on the indenter shaft during immersion IIT were not considered in this study. Furthermore, compared with the 2D Gaussian distribution model used in FE simulations, practical marine immersion often induces non-uniform pitting corrosion and fragile oxide layers [48]. These features exhibit asymmetric micro-asperities and localized material property gradients that complicate indentation contact area determinations compared to homogeneous FE models. In addition, practical immersion IIT are highly sensitive to thermal drift caused by the temperature difference between the fluid and the indenter, as well as structural compliance shifts. Distinguishing micro- and nanoscale material responses from environmental instrument noise remains a primary hurdle in high-precision experimental calibration. Consequently, to bridge this gap, future experimental work will focus on utilizing reference materials with known, stable properties to calibrate the apparatus and incorporate dynamic viscosity calibrations into the load-correction algorithms.

In addition, it is worth noting that there is abundant room for improvement in evaluating the effect of surface roughness on indentation responses. A 2D axisymmetric indentation model can be established by describing the rough surface with sinusoidal curves and employing an axisymmetric self-similar indenter. Several studies have suggested that this model exhibits reasonable applicability and accuracy in revealing the critical influence of surface roughness [28,30]. However, the micromechanical behaviors could be slightly overestimated compared to those of the 3D continuum due to the geometric constraint for out-of-plane material flow. Furthermore, although randomly selecting the indentation site through translation for 3D models is difficult for the 2D axisymmetric assumption, the contact positioning between the indenter and the rough surface can still be determined by the phase shift [29] or user-defined subroutines for *Z*-coordinate values.

## 4. Conclusions

Through a combined theoretical and numerical approach, this study investigated the effects of buoyancy and surface roughness on the mechanical performance of EH36 high-strength steel during immersion instrumented indentation. The findings provide critical insights into how these factors influence IIT results, thereby offering predictive quantitative insights into error ranges during parametric simulations. The main conclusions are summarized as follows:

(1) The comparison studies in *P*-*h* curves of immersion indentation and conventional indentation indicate that the buoyancy could affect the contact stiffness and the indentation load, leading to differences in mechanical properties. As the maximum indentation depth increases, the calculated error for reduced modulus gradually increases from 25.35% to 33.06%. However, the measured values of indentation hardness are almost independent of the indentation depth, and the relative error of ~1.92% can be negligible in practical applications.
(2) Surface roughness has a significant effect on *P*-*h* curves and the dispersity of indentation data, especially at shallow indentations. When the indentation depth is on the same order as the characteristic length scale of surface roughness, unexpectedly higher measured values are observed owing to the hardening effect of surface asperities. As the maximum indentation depth increases for rough surface models, the measured results gradually trend towards the reference values obtained from the smooth surface model.
(3) The combined effects of buoyancy and surface roughness on the dispersity of indentation data exhibit a competitive relationship rather than a monotonous cumulative effect. The measured errors of reduced modulus at roughness values below 0.10 μm are predominantly affected by buoyancy during immersion indentations. With the progressive increase of *Ra* values, surface roughness becomes the dominant factor affecting the error variation. Meanwhile, indentation hardness assessments are mainly affected by surface roughness.

## Data Availability

The data that support the findings of this research are available from the corresponding author upon reasonable request.

## References

[1] Chen M.-T., Pan Y., Ren F., Cao H., Lai M., Zhang J. (2025). Experimental investigation and predictive model on tensile behavior of corroded high-strength steel and butt-welded connections. Thin-Walled Struct..

[2] Wei H., Tang Y., Chen C., Xi P. (2024). Corrosion behavior and microstructure analysis of butt welds of Q690 high strength steel in simulated marine environment. J. Build. Eng..

[3] Liu X., Guo Z., Bai D., Yuan C. (2022). Study on the mechanical properties and defect detection of low alloy steel weldments for large cruise ships. Ocean Eng..

[4] Dong Y., Ai Z., Liu L. (2025). Numerical investigation on the effect of material inhomogeneity of welded joints on the local stress and strain. J. Mar. Sci. Appl..

[5] Pamnani R., Karthik V., Jayakumar T., Vasudevan M., Sakthivel T. (2016). Evaluation of mechanical properties across micro alloyed HSLA steel weld joints using Automated Ball Indentation. Mater. Sci. Eng. A.

[6] Ragu Nathan S., Balasubramanian V., Malarvizhi S., Rao A.G. (2015). Effect of welding processes on mechanical and microstructural characteristics of high strength low alloy naval grade steel joints. Def. Technol..

[7] Ma Z., Huang B., Liu D., Lu F., Zhao H., Ren L. (2022). Full domain surface distributions of micromechanical properties of articular cartilage structure obtained through indentation array. J. Mater. Res. Technol..

[8] Kumar A., Luktuke A., Torbati-Sarraf H., Sinclair D.R., Chawla N. (2025). Investigation of mechanical properties of corrosion products in AA7075-T651 using in situ nanoindentation. Corros. Sci..

[9] Zeiler S., Jelinek A.S., Terziyska V., Schwaiger R., Mitterer C., Brinckmann S., Maier-Kiener V. (2024). A new approach for in situ electrochemical nanoindentation: Side charging as a promising alternative. Acta Mater..

[10] Wang S.B., Wu O.Y., Li S.R., Wang Y.Y., Zhao H.W. (2022). A minimized and efficient low temperature loading device for indentation. Rev. Sci. Instrum..

[11] de Vasconcelos L.S., Xu R., Zhao K. (2017). Operando nanoindentation: A new platform to measure the mechanical properties of electrodes during electrochemical reactions. J. Electrochem. Soc..

[12] Wang X., Chen K., de Vasconcelos L.S., He J., Shin Y.C., Mei J., Zhao K. (2020). Mechanical breathing in organic electrochromics. Nat. Commun..

[13] Duarte M.J., Fang X., Rao J., Krieger W., Brinckmann S., Dehm G. (2021). In situ nanoindentation during electrochemical hydrogen charging: A comparison between front-side and a novel back-side charging approach. J. Mater. Sci..

[14] Song Y., Bhargava B., Stewart D.M., Talin A.A., Rubloff G.W., Albertus P. (2023). Electrochemical-mechanical coupling measurements. Joule.

[15] Chen Z., Diebels S. (2012). Modelling and parameter re-identification of nanoindentation of soft polymers taking into account effects of surface roughness. Comput. Math. Appl..

[16] Charvátová Campbell A., Buršíková V., Martinek J., Klapetek P. (2019). Modeling the influence of roughness on nanoindentation data using finite element analysis. Int. J. Mech. Sci..

[17] Zhu W., Zhang K., Tan J., Liu X., Tu S. (2025). Effects of residual stress and surface roughness on measurement of mechanical properties of IN718 by instrumented indentation testing. J. Mater. Res. Technol..

[18] Wang Z.X., Li L.J., Zhang Z.Y., Ji W., Li M., Zong X.Y., Li C., Wang H., Wang J.B. (2025). Investigation on indentation scaling relationships of ITO thin films considering the indenter tip rounding defect. Mater. Des..

[19] Wang Z., Wang S., Niu Y., Zhao H. (2022). A study of effect factors on thermal drift rates during cryogenic indentation via Taguchi design and finite element method. Mater. Des..

[20] Wang Z., Liu D., Ji W., Li M. (2025). Indentation responses and deformation mechanisms of high-entropy alloy coatings on porous Ti6Al4V substrates: Simulations and dimensional analysis. Mater. Today Commun..

[21] Wang Z., Wang J., Wang W., Niu Y., Li C., Zong X., Zhang J., Zhao H. (2021). Scaling relationships of elastic-perfectly plastic film/coating materials from small scale sharp indentation. Sci. China Technol. Sci..

[22] Zhang Y., Zhou M., Gao W., Liu X., Peng Z. (2026). Trajectory method versus forming method—Influence of turning-induced surface integrity on fatigue performance of V-shaped notch specimens. Measurement.

[23] Wang Z., Zhang M., Li M., Li X., Li L., Zhao H. (2026). Micromechanical properties and deformation damage behaviors of SiCp/Al composites using cryogenic indentation technique. J. Mater. Res. Technol..

[24] Wang S., Xu H., Wang Y., Kong L., Wang Z., Liu S., Zhang J., Zhao H. (2019). Design and testing of a cryogenic indentation apparatus. Rev. Sci. Instrum..

[25] Zong X., Li Y., Wang S., Guan S., Tian L., Li X., Zhao H. (2025). Development of a miniaturized indentation device for extremely low temperatures down to 4.2 K. IEEE Trans. Instrum. Meas..

[26] Wang S., Liu H., Xu L., Du X., Zhao D., Zhu B., Yu M., Zhao H. (2017). Investigations of phase transformation in monocrystalline silicon at low temperatures via nanoindentation. Sci. Rep..

[27] Huang H., Zhao H., Shi C., Zhang L., Wan S., Geng C. (2013). Randomness and statistical laws of indentation-induced pop-out in single crystal silicon. Materials.

[28] Cech V., Lasota T., Palesch E., Lukes J. (2015). The critical influence of surface topography on nanoindentation measurements of a-SiC:H films. Surf. Coat. Technol..

[29] Tang D., Zhao L., Wang H., Li D., Peng Y., Wu P. (2021). The role of rough surface in the size-dependent behavior upon nano-indentation. Mech. Mater..

[30] Li X., Zhang W., Li D., Zhang J., Long B. (2022). Numerical study on the regression method to eliminate the influence of surface morphology on indentation hardness of thin films. Coatings.

[31] Haušild P., Čech J., Materna A., Matějíček J. (2019). Statistical treatment of grid indentation considering the effect of the interface and the microstructural length scale. Mech. Mater..

[32] Marteau J., Bouvier S., Bigerelle M. (2015). Review on numerical modeling of instrumented indentation tests for elastoplastic material behavior identification. Arch. Comput. Methods Eng..

[33] Wheeler J.M., Michler J. (2013). Invited Article: Indenter materials for high temperature nanoindentation. Rev. Sci. Instrum..

[34] Park S.-J., Lee K., Cerik B.C., Choung J. (2019). Ductile fracture prediction of EH36 grade steel based on Hosford–Coulomb model. Ships Offshore Struct..

[35] Park S.-J., Lee K., Cerik B.C., Choung J. (2019). Comparative study on various ductile fracture models for marine structural steel EH36. J. Ocean Eng. Technol..

[36] Xu B., Chen X. (2010). Determining engineering stress–strain curve directly from the load–depth curve of spherical indentation test. J. Mater. Res..

[37] Haynes W.M. (2016). CRC Handbook of Chemistry and Physics.

[38] Lim Y., Ha S. (2023). RufGen: A plug-in for rough surface generation in Abaqus/CAE. SoftwareX.

[39] Li Z., Ye Y., Zhang G., Guan F., Luo J., Wang P., Zhao J., Zhao L. (2023). Research on determining elastic-plastic constitutive parameters of materials from load depth curves based on nanoindentation technology. Micromachines.

[40] Niu J., Tian P., Sun S., Zhang Y., Song G., Song Q., Li Q., Hu N., Li F. (2024). Non-destructive hardness indentation measurement of residual stress on large aerospace forged components at the engineering site based on impact hardness tester. Materials.

[41] Karbasian A., Mahmoudi A.H. (2023). Application of spherical macro-indentation for determination of plastic anisotropy and residual stresses using indentation geometry and inverse analysis. Proc. Inst. Mech. Eng. Part L J. Mater. Des. Appl..

[42] Zhou M., Fan Z., Ma Z., Guo Y., Yang L., Qian L., Sun X. (2017). Effects of flotage on immersion indentation results of bone tissue: An investigation by finite element analysis. Adv. Mater. Sci. Eng..

[43] Kim J.-Y., Kang S.-K., Lee J.-J., Jang J.-i., Lee Y.-H., Kwon D. (2007). Influence of surface-roughness on indentation size effect. Acta Mater..

[44] Böhme L., Keksel A., Ströer F., Bohley M., Kieren-Ehses S., Kirsch B., Aurich J.C., Seewig J., Kerscher E. (2019). Micro hardness determination on a rough surface by using combined indentation and topography measurements. Surf. Topogr.-Metrol. Prop..

[45] Marteau J., Bigerelle M. (2017). Toward an understanding of the effect of surface roughness on instrumented indentation results. J. Mater. Sci..

[46] Zhang T.-Y., Xu W.-H., Zhao M.-H. (2004). The role of plastic deformation of rough surfaces in the size-dependent hardness. Acta Mater..

[47] Chen L., Ahadi A., Zhou J., Ståhl J.-E. (2013). Modeling effect of surface roughness on nanoindentation tests. Procedia CIRP.

[48] Jeffrey R., Melchers R.E. (2007). The changing topography of corroding mild steel surfaces in seawater. Corros. Sci..

